# Effect of Different Omega-6/Omega-3 Polyunsaturated Fatty Acid Ratios on the Formation of Monohydroxylated Fatty Acids in THP-1 Derived Macrophages

**DOI:** 10.3390/biology4020314

**Published:** 2015-04-09

**Authors:** Kathrin Keeren, Dan Huang, Christopher Smyl, Andreas Fischer, Michael Rothe, Karsten-H. Weylandt

**Affiliations:** 1 Department of Medicine, Division of Hepatology, Gastroenterology and Endocrinology, Charité University Medicine Berlin, Campus Virchow-Klinikum, Berlin 13353, Germany; E-Mails: KeerenK@rki.de (K.K.); dan.huang@charite.de (D.H.); christopher.smyl@charite.de (C.S.); andi.fischer@charite.de (A.F.); 2 Lipid Clinic, Experimental and Clinical Research Centre (ECRC), Charité University Medicine and Max Delbrueck Center for Molecular Medicine, Berlin 13353, Germany; 3 Lipidomix GmbH, Berlin 13125, Germany; E-Mail: michael.rothe@lipidomix.de

**Keywords:** omega-3, omega-6, LC/ESI-MS/MS, lipid mediators, THP-1 macrophages, 17-HDHA, 18-HEPE, 14-HDHA, HETE, HEPE, HDHA

## Abstract

Omega-6 and omega-3 polyunsaturated fatty acids (n-6 and n-3 PUFA) can modulate inflammatory processes. In western diets, the content of n-6 PUFA is much higher than that of n-3 PUFA, which has been suggested to promote a pro-inflammatory phenotype. The aim of this study was to analyze the effect of modulating the n-6/n-3 PUFA ratio on the formation of monohydroxylated fatty acid (HO-FAs) derived from the n-6 PUFA arachidonic acid (AA) and the n-3 PUFAs eicosapentaenoic acid (EPA) and docosahexaenoic acid (DHA) in THP-1 macrophages by means of LC-MS. Lipid metabolites were measured in THP-1 macrophage cell pellets. The concentration of AA-derived hydroxyeicosatetraenoic acids (HETEs) was not significantly changed when incubated THP-1 macrophages in a high AA/(EPA+DHA) ratio of 19/1 *vs.* a low ratio AA/(EPA+DHA) of 1/1 (950.6 ± 110 ng/mg *vs.* 648.2 ± 92.4 ng/mg, *p* = 0.103). Correspondingly, the concentration of EPA-derived hydroxyeicosapentaenoic acids (HEPEs) and DHA-derived hydroxydocosahexaenoic acids (HDHAs) were significantly increased (63.9 ± 7.8 ng/mg *vs.* 434.4 ± 84.3 ng/mg, *p* = 0.012 and 84.9 ± 18.3 ng/mg *vs.* 439.4 ± 82.7 ng/mg, *p* = 0.014, respectively). Most notable was the strong increase of 18-hydroxyeicosapentaenoic acid (18-HEPE) formation in THP-1 macrophages, with levels of 170.9 ± 40.2 ng/mg protein in the high n-3 PUFA treated cells. Thus our data indicate that THP-1 macrophages prominently utilize EPA and DHA for monohydroxylated metabolite formation, in particular 18-HEPE, which has been shown to be released by macrophages to prevent pressure overload-induced maladaptive cardiac remodeling.

## 1. Introduction

A state of chronic subclinical inflammation has been proposed to play a significant role in the pathogenesis of a multitude of human diseases, including atherosclerosis, rheumatoid arthritis, non-alcoholic fatty liver disease and the development of premalignant lesions in the gastrointestinal tract [1,2,3]. Lipid mediators derived from polyunsaturated fatty acids (PUFAs) constitute major regulators of inflammation with omega-6 (n-6) PUFA metabolites being mostly pro-inflammatory whereas omega-3 (n-3) PUFA derivatives have been implicated in the modulation of macrophage function and alleviation of chronic inflammatory responses [4,5].

The n-3 PUFA eicosapentaenoic acid (EPA) and docosahexaenoic acid (DHA) were shown to exert effects through EPA-derived E-series and DHA-derived D-series resolvins, protectins and maresins [6,7,8,9,10]. It has been suggested that resolvins, protectins and maresins, collectively termed specialized pro-resolving mediators (SPMs), are involved in the regulation of inflammatory response [6,8,11,12]. They are thought to limit polymorphonuclear neutrophil infiltration, enhance macrophage clearance of apoptotic cells and switch macrophages from the pro-inflammatory phenotype M1 to the M2 phenotype, which is associated with anti-inflammatory and restorative macrophage functions [13]. These events might be crucial for the resolution of inflammation [14,15]. Macrophages also play a role in wound healing and organ regeneration via SPMs [6,16].

E-resolvins are hydroxylated derivatives of the parent n-3 PUFA eicosapentaenoic acid (EPA), and D-resolvins and protectin D1, as well as maresins, are the hydroxylation products of docosahexaenoic acid (DHA) (reviewed in [10,17]). Biochemical pathways of resolvin and protectin formation involve common monohydroxylated markers, which are the 15-lipoxygenase (15-LOX) metabolite 17-hydroxydocosahexaenoic acid (17-HDHA) for the DHA-derived resolvins, the 12-lipoxygenase (12-LOX) metabolite 14-hydroxydocosahexaenoic acid (14-HDHA) for the DHA-derived maresins and 18-hydroxyeicosapentaenoic acid (18-HEPE) for the EPA-derived compounds, for which the enzymatic synthesis pathway is currently not well established.

In animal experimental models, 17-HDHA, RvD1 and RvD2 reduced neutrophilic infiltration, pain and tissue damage, stimulated murine dermal healing and re-epithelialization [18,19,20]. Maresins are synthesized from DHA by the 12-LOX enzyme [21,22]. One of these molecules, maresin-1 (MaR1) was recently found to dampen the pro-inflammatory response to organic dust in bronchial epithelial cells [23], attenuate mouse colitis [7] and enhance human macrophage phagocytosis [24].

Using animal models, work in our laboratory explored the role of n-3 PUFA and their metabolites in the dampening of inflammation and inflammation-related tumorigenesis. In a mouse model of chemically induced colitis we observed the formation of high levels of 17-HDHA [25], whereas other work [26] demonstrated inflammation-dampening and resolution-promoting effects of 17-HDHA. These data are in agreement with the observations made by other groups [9,27,28,29]. Using 17-HDHA in an *in vitro* phagocytosis model in murine RAW 264.7 macrophages we were able to show increased phagocytosis due to this compound [25], which is consistent with its role in the resolution process. Furthermore we found decreased TNF-α expression and increased expression of the scavenger receptor A in these cells when treated with 17-HDHA [26].

17-HDHA, as well as 18-HEPE was able to suppress TNF-α secretion from RAW 264.7 macrophages in another set of experiments [30]. This previous study also documented a highly significant increase in the formation of 17-HDHA and 18-HEPE in the context of a chemically induced liver tumor model in the fat-1 transgenic mouse. We were also able to demonstrate that increasing the endogenous content of omega-3 fatty acids in the fat-1 mouse raises the blood generation capacity of 17-HDHA as well as that of 18-HEPE derived from EPA [10]. 18-HEPE was recently found to inhibit macrophage-mediated pro-inflammatory activation of cardiac fibroblasts [31]*.*

While these experiments have so far focused on mice and murine macrophages, our data from human blood samples indicate the presence of a wide spectrum of HO-FAs also in human blood, with a predominance of 5-, 12- and 15-lipoxygenase products [32]. Lipoxygenases (LOXes) mediate the oxygenation of free fatty acids, forming a multitude of mono-and poly-hydroxy fatty acids: e.g., AA produces hydroxyeicosatetraenoic acids (HETEs), EPA generates hydroxyeicosapentaenoic acids (HEPEs), DHA derives hydroxydocosahexaenoic acids (HDHAs) [33,34]; LOX enzymes are crucial in the formation of resolvins, protectins and maresins.

It was therefore the aim of this study to assess the formation of these HO-FAs (HETEs, HEPEs and HDHAs) in the human THP-1 cell line differentiated into a macrophage phenotype. THP-1 macrophages have been used extensively in the assessment of omega-3 PUFA and studies have shown their effect on fatty acid metabolism and nuclear receptor genes [35,36], possibly leading to anti-inflammatory effects. However, to our knowledge, no analysis of HO-FAs from omega-3 and omega-6 PUFA has been performed yet in this model.

In this study we assess the formation of lipid metabolite and mediator compounds derived from the n-6 PUFA AA, the n-3 PUFA EPA and DHA in human THP-1 macrophages using lipidomics (LC-MS/MS) technology in context of two different AA/(EPA+DHA) ratios, 19/1 *vs.* 1/1, in attempt to model the high n-6/n-3 PUFA ratio present in the western diet as compared to more traditional diets with a balanced n-6/n-3 PUFA [37]. The data presented here show that an increased supplementation of the n-3 PUFA EPA and DHA leads to significant increases of n-3 PUFA derived HEPEs and HDHAs in THP-1 macrophage cells with the most prominent effect on 18-HEPE.

## 2. Experimental Section

### 2.1. Chemicals and Biological Materials

The human monocytic cell line THP-1 was from the ATCC (TIB 202), Roswell Park Memorial Institute medium 1640 (RPMI 1640), fetal calf serum (FCS), L-glutamine, penicillin/streptomycin were purchased from Life Technologies. Phorbol-12-myristate-13-acetate (PMA) was from Sigma Aldrich (P8139, Taufkirchen, Germany). Bovine serum albumin (BSA) was purchased from Merck (Darmstadt, Germany). AA, EPA, DHA and internal standards were purchased from Cayman Chemicals (Ann Arbor, MI, USA). Materials used for Solid phase Extraction (SPE), such as sodium acetate, acetic acid ethyl ester, acetic acid and n-hexane were obtained from Carl Roth (Karlsruhe, Germany) and methanol bought from Merck (Darmstadt, Germany). Butylhydroxytoluol (BHT, 2,6-Di-tert-butyl-4-methylphenol) at 99% was from Acros Organics (Geel, Belgium) and Bond Elute Certify II columns from Varian (Palo Alto, CA, USA). Other solvents such as methanol, LC-MS-grade, and acetonitrile HPLC gradient grade, were from Fisher Scientific (Loughborough, UK).

### 2.2. Cell Culture

THP-1 cells were maintained in RPMI 1640 supplemented with 2 mM L-glutamine and 10% v/v FCS, with 100 U/mL penicillin and 100 µg/mL streptomycin at 37 °C in a humidified atmosphere of 95% air and 5% CO_2_. THP-1 (4 × 10^6^/25 cm^2^ culture flask) cells were differentiated into macrophages by incubation with PMA (200 nM, 72 h). Cells were serum deprived (10% FCS replaced by 1 mg/mL fatty acid free BSA) for 24 h prior to supplementation with two different n-6/n-3 ratios or vehicle (Ethanol) for 6 h. A high n-6/n-3 ratio was mimicked by 95 µM AA + 2.5 µM EPA + 2.5 µM DHA and a low ratio by 50 µM AA + 25 µM EPA + 25 µM DHA. Fatty acids were dissolved in ethanol, which was evaporated by a gentle N_2_ stream when preparing the actual incubation media and resolved in 10% FCS containing RPMI 1640. Evaporation was performed in order to avoid the toxic effects of ethanol when exposing the cells to the fatty-acid containing media. The same incubations were carried out with and without cells. Cell-free culture medium was sampled after 6 h incubation. Cells were harvested by scratching after 6 h incubation and stored as cell pellets. All samples were immediately frozen at −80 °C until further analysis. All experiments were carried out in triplicates.

### 2.3. Sample Preparation

Internal standards (IS) consisting of 15-HETE-d_8_ (10 ng), LTB_4_-d_4_ (10 ng) and ice-cold methanol containing BHT (0.1%) were add to cell pellets. 300 µL sodium hydroxide (10 M) were added. The whole sample was stored in a drying oven at 60 °C for 30 min to saponification.

After neutralization with 300 µL acetic acid (58%) the pH was adjusted with 3 mL 1 M sodium acetate buffer containing 5% v/v methanol at pH = 6.

After centrifugation (10 min, RT, 3500 rpm) an aliquot of 50 µL was taken for determination of total protein using a Modified Lowry Protein Assay Kit (Thermo Scientific Pierce, Loughborough, UK).

In order to exclude an effect of the hydrolysis reaction on protein measurement control measurements were performed using albumin solutions with and without alkaline hydrolysis yielding 289.5 ± 36.4 µg/mL *vs.* 283.0 ± 43.7 µg/mL (*n =* 12) with no significant difference. All available material per sample was processed for analysis and the total protein quality of samples were between 230 to 410 µg. The SPE column (Bond-Elut Certify II, Agilent Technolgies, Santa Clara, CA, USA) were preconditioned with 3 mL methanol, followed by 3 mL of 0.1 mol/L sodium acetate buffer containing 5% methanol (pH 6). The SPE-columns were then washed with 3 mL methanol/H_2_O (50/50, v/v). For elution 2.0 mL of n-hexane: ethyl acetate 25:75 with 1% acetic acid was used. The extraction was performed with a SUPELCO Visiprep manifold. The eluate was evaporated on a heating block at 40 °C under a stream of nitrogen to obtain a solid residue. The residues were dissolved in 70 µL acetonitrile and stored at −20 °C until LC/ESI-MS/MS analysis was performed.

### 2.4. LC/ESI-MS/MS

The samples were analyzed using an Agilent 1200 HPLC system with binary pump, autosampler and column thermostat with a Zorbax Stable Bond 3.5 µM, 2.1 × 150 mm column using a solvent system of aqueous formic acid (0.1%) and acetonitrile. The elution gradient was started with 10% acetonitrile, which was increased within 10 min to 90% and held there for 10 min. The flow rate was set at 0.4 mL/min, the injection volume was 7.5 µL. The HPLC was coupled with an Agilent 6460 Triplequad mass spectrometer with electrospray ionization source. Analysis of lipid mediators was performed with Multiple Reaction Monitoring in negative mode. Limits of detection (LOD) and limits of quantitation (LOQ) for the assayed compounds are given in the supplementary Table S1. Mean recovery of internal standards (IS) was 78% for 15-HETE-d_8_ (used for the quantitation of all HETEs, HEPEs and HDHAs) and 40% for LTB_4_-d_4_ (used for the quantitation of resolvin D1 and lipoxin A4. Given the low recovery of the IS LTB4-d4 we chose not to include resolvin D1 and Lipoxin A4 measurements in the presented analyses.

### 2.5. Statistical Analysis

All experiments were repeated three times and data are presented as mean ± SEM. Data were tested for normal distribution using Shapiro-Wilk statistics. For the analytes for which the null-hypothesis was accepted we assumed normal distribution subsequently used t-test statistics. For the metabolites where normal distribution testing rejected the null hypothesis (11-HETE, 11-HDHA and 13-HDHA) we performed Mann-Whitney U testing. Statistical analyses were made using SPSS 22.0 for windows (IBM Corporation, NY, USA), with *p* < 0.05 considered statistically significant.

## 3. Results and Discussion

**Figure 1:**
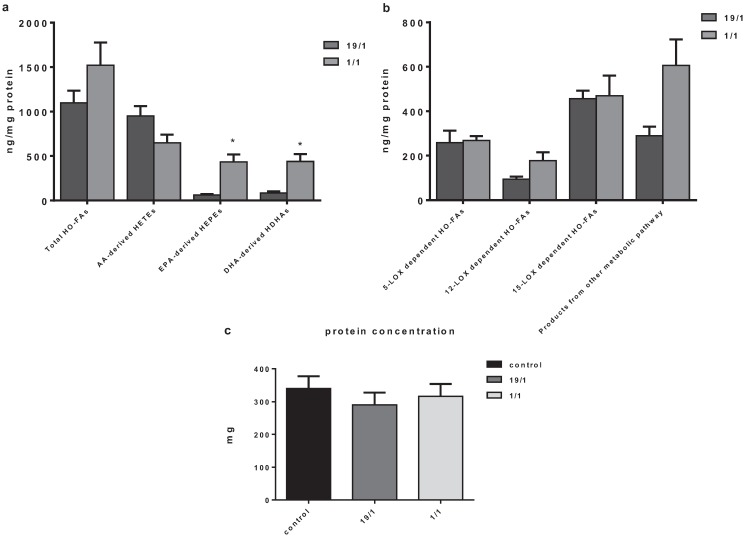
Comparison of metabolites derived from arachidonic acid (AA)/eicosapentaenoic acid (EPA)/docosahexaenoic acid (DHA), in response to incubation of THP-1 macrophages with a high ratio of AA/(EPA+DHA) (19/1) *vs.* a low ratio of AA/(EPA+DHA) (1/1). (**a**) Total monohydroxylated fatty acids (HO-Fas) and those derived from AA/EPA/DHA respectively; (**b**) HO-FAs formed by combined action of 5-,12-, 15- Lipoxygenase (LOX), and the other metabolic pathways or autooxidation. * *p* < 0.05, ** *p* < 0.01 1/1 *vs.* 19/1 ratio; (**c**) There was no significant difference in THP-1 macrophage growth between the two groups during the 6 h polyunsaturated fatty acid (PUFA) incubation as evidenced by similar total protein content in the cell pellets (*p* = 0.7 for 19/1 *vs.* 1/1).

Incubating THP-1 macrophages in medium with a total concentration of 100 µM PUFA (95 µM AA and 2.5 µM EPA and DHA, respectively, reflecting an (AA/(EPA+DHA) ratio of 19/1) led to the formation of a total of 1099.4 ± 135.7 ng/mg of the assayed HETEs, HEPEs and HDHAs that could be derived from AA, EPA and DHA. Reducing the AA/EPA+DHA ratio to 1/1 by using a combination of 50 µM AA and 25 µM EPA and DHA, respectively, did not result in a significant change of the sum of the HETEs, HEPEs and HDHAs (mean concentration 1522.1 ± 256 ng/mg; *p* = 0.218 *vs.* high AA/(EPA+DHA) ratio). The concentration of compounds derived from EPA and DHA (HEPEs and HDHAs) significantly increased from 63.9 ± 7.8 ng/mg to 434.4 ± 84.3 ng/mg and 84.9 ± 18.3 ng/mg to 439.4 ± 82.7 ng/mg, respectively, under these conditions (Figure 1a).

In contrast, the concentration of AA-derived metabolites was not significantly reduced (950.6 ± 110 ng/mg and 648.2 ± 92.4 ng/mg with 95 µM and 50 µM AA, respectively). When calculating the relative contribution of n-6 and n-3 PUFA derivatives, we found that a ratio of AA to n-3 PUFA of 19/1 resulted in a ratio of AA- (HETEs) to EPA- and DHA-derived metabolites (HEPEs and HDHAs) of 6.5/1, indicating higher use of EPA and DHA for monohydroxylation at low concentrations. This preferential utilization of n-3 PUFA was maintained although to a lesser extent with higher concentrations, as a 1/1 ratio of AA/(EPA+DHA) yielded a ratio of HETEs/(HEPEs+HDHAs) of 0.8/1. Figure 1b demonstrates that these changes were not accompanied by differential activation of LOX pathways as the total amount of 5-, 12- and 15-LOX-dependent HO-FAs derived from AA (5-,12-,8- and 15-HETE), EPA (5-,12-,8- and 15-HEPE) and DHA (4-,7-,11-,14-,10- and 17-HDHA) did not differ significantly between groups.

Different PUFAs could have different effects on cell growth. While we did not directly measure cell growth or proliferation, protein measurements were performed for each sample. The total protein content of samples was between 230 to 410 µg; however, there was no significant difference between the two groups, indicating that cell growth did not differ significantly between groups. There were also no differences to THP-1 macrophages grown for 6 h without addition of PUFA (Figure 1c).

**Figure 2:**
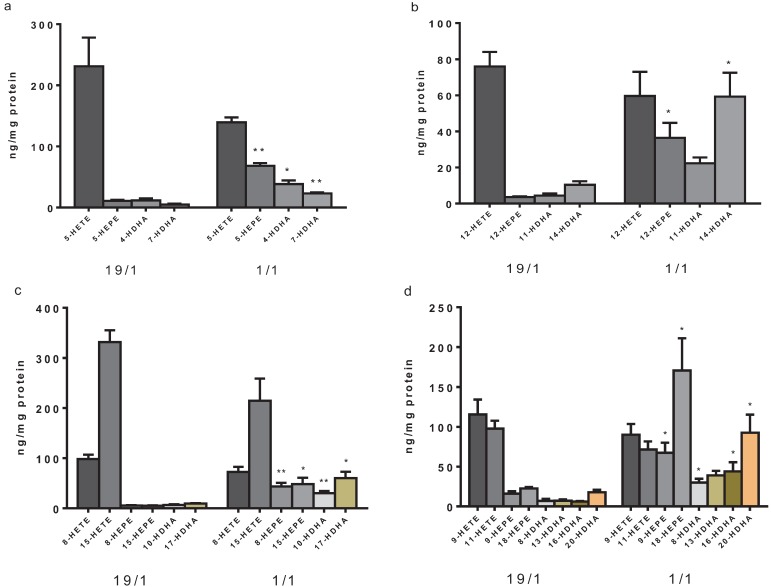
Metabolites that can be contributed to different LOX enzymes in a high ratio of AA/(EPA+DHA) (19/1) *vs.* low ratio of AA/(EPA+DHA) (1/1). (**a**) metabolites that can be formed by 5-LOX; (**b**) metabolites that can be formed by 12-LOX; and (**c**) metabolites that can be formed by 15-LOX, (**d**) metabolites not attributable to specific enzymatic or other metabolic pathways. * *p* < 0.05, ** *p* < 0.01 1/1 *vs.* 19/1 ratio.

We also analyzed the concentration of single PUFA metabolites that could be generated by different LOX pathways [38]. However, given that our method does not allow for chiral differentiation of metabolites, this study was not able to further discriminate between enzymatic and non-enzymatic (autooxidation) contribution to formation of these metabolites. In line with the overall metabolite concentrations reported above, we found that the concentration of AA derivatives generated by the 5-LOX, 12-LOX and 15-LOX pathways did not significantly differ between groups with a 19/1 and 1/1 ratio of AA/(EPA+DHA) (Figure 2). Conversely, n-3 PUFA metabolites significantly increased in the 1/1 group. The ratio of AA-derivatives to EPA- and DHA-derivatives changed from 8.8 to 1.1 for 5-LOX (metabolizing AA to 5-HETE, EPA to 5-HEPE and DHA to 4-HDHA and 7-HDHA; Figure 2a) and 4.3 to 0.5 for 12-LOX (metabolizing AA to 12-HETE, EPA to 12-HEPE and DHA to 11-HDHA and 14-HDHA; Figure 2b). For 15-LOX we found that the ratio of AA metabolites (8-HETE and 15-HETE) to EPA (8-HEPE and 15-HEPE) and DHA (10-HDHA and 17-HDHA) derivatives decreased from 16.6 to 1.6 upon increasing the concentration of the n-3 PUFA (Figure 2c). In addition to metabolites generated via distinct LOX pathways, the concentration of compounds that have not been shown to be products of known enzymatic activities so far and are currently mostly judged as autooxidation products were analyzed. The concentration of 9- and 11-HETE derived from AA was not significantly changed when incubated in medium with a high *vs.* a low AA/(EPA+DHA) ratio (19/1 and 1/1). In contrast, incubation with low AA/(EPA+DHA) medium led to significantly increased formation of 9- and 18-HEPE (derived from EPA) as well as 8-, 16- and 20-HDHA (derived from DHA) in THP-1 macrophage cells (Figure 2d).

As compared to THP-1 macrophages just grown in normal media without PUFA supplementation, levels of HETEs, HEPEs and HDHAs were very substantially increased by PUFA treatments (Supplementary Figure S1). An important finding was also the, compared to the results in the cell pellets, low unspecific formation of HETEs, HEPEs and HDHAs in medium plus PUFA controls incubated without cells, as significant unspecific formation of these metabolites has been shown before [39,40], albeit with longer incubation periods of 24 h as compared to the 6 h incubation used here (Supplementary Figure S2).

The monohydroxylated metabolites 14-HDHA, 17-HDHA and 18-HEPE are pathway indicators of resolvins, protectins and maresins, and the data presented here demonstrate formation of these crucial metabolites in THP-1 macrophages. Most prominently, these data indicate the prominent formation of 18-HEPE in the context of high EPA levels. Currently it is believed that EPA is converted to 18-HEPE by autooxidation, by aspirin-acetylated COX-2 or by cytochrome P450 monooxygenases [10,41,42,43], however, detailed cytochrome P450 pathways involved are not fully elucidated. 18-HEPE can be further metabolized by 5-LOX and 12/15-LOX to resolvin E1 (RvE1), resolvin E2 (RvE2), and resolvin E3 (RvE3). These compounds are anti-inflammatory, promote inflammation resolution and assist in maintenance of tissue homeostasis [11,29,41]. Interestingly however, 18-HEPE was recently found to inhibit macrophage-mediated pro-inflammatory activation of cardiac fibroblasts both *in vitro* and *in vivo*, whereas in this recent study no such effects were observed for the resolvins (RvE1-3) derived from 18-HEPE [31]. This biologically active role of 18-HEPE itself is in agreement with our previous data showing *in vitro* effects of 18-HEPE [30]. In this study, within the limitation of the detection limits and low IS recovery for LTB4-d4 observed here, resolvin D1, lipoxin A4, and 10,17-DiHDHA were not detected in the cell pellet samples.

In this experiment we chose these two different ratios of n-6/n-3 PUFA solutions based on the PUFA contents in Western diet [37] as compared to a balanced n-6/n-3 ratio. Mixtures of AA, EPA + DHA were used in order to mimic dietary n-6/n-3 ratios *in vitro*. As the long-chain C20 PUFA arachidonic acid and eicosapentaenoic acid, as well as the C22 PUFA docosahexaenoic acid, as well as their metabolites, have been shown to have biologically more potent effects than those of the C18 PUFAs linoleic acid or alpha-linolenic acid the study presented here focused on AA, EPA and DHA.

Future experiments should focus to elucidate mechanisms behind the observed formation of lipid metabolites. While, as outlined above, the measurements performed here do not allow to contribute specific lipid metabolites to specific enzymatic pathways, we believe that LOX enzymes play a role in the formation of the monohydroxylated PUFA metabolites assayed here, as it is known that THP-1 cells express 5- and 15-LOX [44,45]. The future work should address the expression levels of these enzymes. A total PUFA concentration of 100 µM in medium containing serum with its high albumin content was chosen to allow for fatty acid concentrations comparable to those observed in human sera. It was shown in the past that fatty acid uptake into the cells is very rapid under these conditions (reviewed in [46]).

This study characterized lipid metabolite formations by the THP-1 macrophages under resting, baseline conditions. Stimulation of the macrophages would probably lead to change in the lipid metabolite profiles. A thorough characterization of stimulation protocols of these cells under conditions of high and low n-6/n-3 PUFA ratios will be part of future studies.

## 4. Conclusions

The data presented here reveal that EPA- and DHA-enriched THP-1 macrophages synthesized significant quantities of HEPEs and HDHAs. The 18-HEPE levels in the cells incubated with 25 µM EPA was higher than that of all other assayed metabolites except 5- and 15-HETE. Together with the data by Endo *et al.* [31], and a recent human study with n-3 PUFA supplementation [47] also showing 18-HEPE as prominent EPA metabolite, the *in vitro* data presented here confirm 18-HEPE as main metabolite derived from EPA. The finding of this study, that EPA and DHA may be important substrates for metabolism via LOX-pathways, will need further validation by experiments aiming at the analysis of metabolites in the presence and absence of the respective LOX enzymes, e.g., by performing siRNA studies in THP-1 macrophages.

## Supplementary Files

Supplementary File 1

## References

[1] Santos M.J., Fonseca J.E. (2009). Metabolic syndrome, inflammation and atherosclerosis-the role of adipokines in health and in systemic inflammatory rheumatic diseases. Acta Reumatol. Port..

[2] Filkova M., Haluzik M., Gay S., Senolt L. (2009). The role of resistin as a regulator of inflammation: Implications for various human pathologies. Clin. Immunol..

[3] Elinav E., Nowarski R., Thaiss C.A., Hu B., Jin C., Flavell R.A. (2013). Inflammation-induced cancer: Crosstalk between tumours, immune cells and microorganisms. Nat. Rev. Cancer.

[4] Claria J., Gonzalez-Periz A., Lopez-Vicario C., Rius B., Titos E. (2011). New insights into the role of macrophages in adipose tissue inflammation and fatty liver disease: Modulation by endogenous omega-3 fatty acid-derived lipid mediators. Front. Immunol..

[5] Miles E.A., Calder P.C. (2012). Influence of marine n-3 polyunsaturated fatty acids on immune function and a systematic review of their effects on clinical outcomes in rheumatoid arthritis. Br. J. Nutr..

[6] Serhan C.N. (2014). Pro-resolving lipid mediators are leads for resolution physiology. Nature.

[7] Marcon R., Bento A.F., Dutra R.C., Bicca M.A., Leite D.F., Calixto J.B. (2013). Maresin 1, a proresolving lipid mediator derived from omega-3 polyunsaturated fatty acids, exerts protective actions in murine models of colitis. J. Immunol..

[8] Serhan C.N., Chiang N. (2013). Resolution phase lipid mediators of inflammation: Agonists of resolution. Curr. Opin. Pharmacol..

[9] Bento A.F., Claudino R.F., Dutra R.C., Marcon R., Calixto J.B. (2011). Omega-3 fatty acid-derived mediators 17(r)-hydroxy docosahexaenoic acid, aspirin-triggered resolvin D1 and resolvin D2 prevent experimental colitis in mice. J. Immunol..

[10] Weylandt K.H., Chiu C.Y., Gomolka B., Waechter S.F., Wiedenmann B. (2012). Omega-3 fatty acids and their lipid mediators: Towards an understanding of resolvin and protectin formation. Prostaglandins Lipid Med..

[11] Schwab J.M., Chiang N., Arita M., Serhan C.N. (2007). Resolvin E1 and protectin D1 activate inflammation-resolution programmes. Nature.

[12] Serhan C.N., Yacoubian S., Yang R. (2008). Anti-inflammatory and proresolving lipid mediators. Ann. Rev. Pathol..

[13] Biswas S.K., Mantovani A. (2010). Macrophage plasticity and interaction with lymphocyte subsets: Cancer as a paradigm. Nat. Immunol..

[14] Nathan C. (2002). Points of control in inflammation. Nature.

[15] Tabas I., Glass C.K. (2013). Anti-inflammatory therapy in chronic disease: Challenges and opportunities. Science.

[16] Lucas T., Waisman A., Ranjan R., Roes J., Krieg T., Muller W., Roers A., Eming S.A. (2010). Differential roles of macrophages in diverse phases of skin repair. J. Immunol..

[17] Serhan C.N. (2005). Novel eicosanoid and docosanoid mediators: Resolvins, docosatrienes, and neuroprotectins. Curr. Opin. Clin. Nutr. Metab. Care.

[18] Wang S.B., Hu K.M., Seamon K.J., Mani V., Chen Y., Gronert K. (2012). Estrogen negatively regulates epithelial wound healing and protective lipid mediator circuits in the cornea. FASEB J..

[19] Tang Y., Zhang M.J., Hellmann J., Kosuri M., Bhatnagar A., Spite M. (2013). Proresolution therapy for the treatment of delayed healing of diabetic wounds. Diabetes.

[20] Spite M., Claria J., Serhan C.N. (2014). Resolvins, specialized proresolving lipid mediators, and their potential roles in metabolic diseases. Cell Metab..

[21] Dalli J., Zhu M., Vlasenko N.A., Deng B., Haeggstrom J.Z., Petasis N.A., Serhan C.N. (2013). The novel 13s,14s-epoxy-maresin is converted by human macrophages to maresin 1 (MaR1), inhibits leukotriene a4 hydrolase (lta4h), and shifts macrophage phenotype. FASEB J..

[22] Serhan C.N., Yang R., Martinod K., Kasuga K., Pillai P.S., Porter T.F., Oh S.F., Spite M. (2009). Maresins: Novel macrophage mediators with potent antiinflammatory and proresolving actions. J. Exp. Med..

[23] Nordgren T.M., Heires A.J., Wyatt T.A., Poole J.A., LeVan T.D., Cerutis D.R., Romberger D.J. (2013). Maresin-1 reduces the pro-inflammatory response of bronchial epithelial cells to organic dust. Respir. Res..

[24] Deng B., Wang C.W., Arnardottir H.H., Li Y., Cheng C.Y., Dalli J., Serhan C.N. (2014). Maresin biosynthesis and identification of maresin 2, a new anti-inflammatory and pro-resolving mediator from human macrophages. PLOS ONE.

[25] Kohnke T., Gomolka B., Bilal S., Zhou X., Sun Y., Rothe M., Baumgart D.C., Weylandt K.H. (2013). Acetylsalicylic acid reduces the severity of dextran sodium sulfate-induced colitis and increases the formation of anti-inflammatory lipid mediators. BioMed Res. Int..

[26] Chiu C.Y., Gomolka B., Dierkes C., Huang N.R., Schroeder M., Purschke M., Manstein D., Dangi B., Weylandt K.H. (2012). Omega-6 docosapentaenoic acid-derived resolvins and 17-hydroxydocosahexaenoic acid modulate macrophage function and alleviate experimental colitis. Inflamm. Res..

[27] Gonzalez-Periz A., Planaguma A., Gronert K., Miquel R., Lopez-Parra M., Titos E., Horrillo R., Ferre N., Deulofeu R., Arroyo V. (2006). Docosahexaenoic acid (DHA) blunts liver injury by conversion to protective lipid mediators: Protectin d1 and 17s-hydroxy-DHA. FASEB J..

[28] Lima-Garcia J.F., Dutra R.C., da Silva K., Motta E.M., Campos M.M., Calixto J.B. (2011). The precursor of resolvin D series and aspirin-triggered resolvin D1 display anti-hyperalgesic properties in adjuvant-induced arthritis in rats. Br. J. Pharmacol..

[29] Neuhofer A., Zeyda M., Mascher D., Itariu B.K., Murano I., Leitner L., Hochbrugger E.E., Fraisl P., Cinti S., Serhan C.N. (2013). Impaired local production of proresolving lipid mediators in obesity and 17-HDHA as a potential treatment for obesity-associated inflammation. Diabetes.

[30] Weylandt K.H., Krause L.F., Gomolka B., Chiu C.Y., Bilal S., Nadolny A., Waechter S.F., Fischer A., Rothe M., Kang J.X. (2011). Suppressed liver tumorigenesis in fat-1 mice with elevated omega-3 fatty acids is associated with increased omega-3 derived lipid mediators and reduced TNF-alpha. Carcinogenesis.

[31] Endo J., Sano M., Isobe Y., Fukuda K., Kang J.X., Arai H., Arita M. (2014). 18-HEPE, an n-3 fatty acid metabolite released by macrophages, prevents pressure overload-induced maladaptive cardiac remodeling. J. Exp. Med..

[32] Gomolka B., Siegert E., Blossey K., Schunck W.H., Rothe M., Weylandt K.H. (2011). Analysis of omega-3 and omega-6 fatty acid-derived lipid metabolite formation in human and mouse blood samples. Prostaglandins Lipid Med..

[33] Kuhn H., O’Donnell V.B. (2006). Inflammation and immune regulation by 12/15-lipoxygenases. Progress Lipid Res..

[34] Nicolaou A., Mauro C., Urquhart P., Marelli-Berg F. (2014). Polyunsaturated fatty acid-derived lipid mediators and T cell function. Front. Immunol..

[35] Gillies P.J., Bhatia S.K., Belcher L.A., Hannon D.B., Thompson J.T., Vanden Heuvel J.P. (2012). Regulation of inflammatory and lipid metabolism genes by eicosapentaenoic acid-rich oil. J. Lipid Res..

[36] Lee J.Y., Hwang D.H. (2002). Docosahexaenoic acid suppresses the activity of peroxisome proliferator-activated receptors in a colon tumor cell line. Biochem. Biophys. Res. Commun..

[37] Simopoulos A.P. (2011). Importance of the omega-6/omega-3 balance in health and disease: Evolutionary aspects of diet. World Rev. Nutr. Diet..

[38] Norris P.C., Dennis E.A. (2012). Omega-3 fatty acids cause dramatic changes in TLR4 and purinergic eicosanoid signaling. Proc. Natl. Acad. Sci. USA.

[39] Jaudszus A., Gruen M., Watzl B., Ness C., Roth A., Lochner A., Barz D., Gabriel H., Rothe M., Jahreis G. (2013). Evaluation of suppressive and pro-resolving effects of EPA and DHA in human primary monocytes and T-helper cells. J. Lipid Res..

[40] Ostermann A.I., Willenberg I., Weylandt K.H., Schebb N.H. (2014). Development of an online-SPE-LC-MS/MS method for 26 hydroxylated polyunsaturated fatty acids as rapid targeted metabolomics approach for the LOX, CYP, and autoxidation pathways of the arachidonic acid cascade. Chromatographia.

[41] Isobe Y., Arita M., Matsueda S., Iwamoto R., Fujihara T., Nakanishi H., Taguchi R., Masuda K., Sasaki K., Urabe D. (2012). Identification and structure determination of novel anti-inflammatory mediator resolvin E3, 17,18-dihydroxyeicosapentaenoic acid. J. Biol. Chem..

[42] Serhan C.N., Brain S.D., Buckley C.D., Gilroy D.W., Haslett C., O’Neill L.A., Perretti M., Rossi A.G., Wallace J.L. (2007). Resolution of inflammation: State of the art, definitions and terms. FASEB J..

[43] Serhan C.N. (2007). Resolution phase of inflammation: Novel endogenous anti-inflammatory and proresolving lipid mediators and pathways. Ann. Rev. Immunol..

[44] Weibel G.L., Joshi M.R., Wei C., Bates S.R., Blair I.A., Rothblat G.H. (2009). 15(S)-lipoxygenase-1 associates with neutral lipid droplets in macrophage foam cells: Evidence of lipid droplet metabolism. J. Lipid Res..

[45] Riddick C.A., Ring W.L., Baker J.R., Hodulik C.R., Bigby T.D. (1997). Dexamethasone increases expression of 5-lipoxygenase and its activating protein in human monocytes and THP-1 cells. Eur. J. Biochem..

[46] McArthur M.J., Atshaves B.P., Frolov A., Foxworth W.D., Kier A.B., Schroeder F. (1999). Cellular uptake and intracellular trafficking of long chain fatty acids. J. Lipid Res..

[47] Fischer R., Konkel A., Mehling H., Blossey K., Gapelyuk A., Wessel N., von Schacky C., Dechend R., Muller D.N., Rothe M. (2014). Dietary omega-3 fatty acids modulate the eicosanoid profile in man primarily via the CYP-epoxygenase pathway. J. Lipid Res..

