# Supplementary material for: Effect of Different Omega-6/Omega-3 Polyunsaturated Fatty Acid Ratios on the Formation of Monohydroxylated Fatty Acids in THP-1 Derived Macrophages
Source: Biology (Basel). 2015 Apr 9;4(2):314–26. doi: 10.3390/biology4020314 (PMC4498302; doi:10.3390/biology4020314)
Supplement: Supplementary File 1 [file biology-04-00314-s001.pdf]

Supplemental Materials

Table S1. Limit of quantitation (LOQ) for the metabolites assayed. The LOQ is calculated as limit of detection (LOD) × 3 (determined as signal to noise, RMS, for smoothed signals).

Target	LOQ [pg per sample]
5-HEPE	3
12-HEPE	31
15-HEPE	4
18-HEPE	10
5-HETE	3
12-HETE	21
15-HETE	2
4-HDHA	16
14-HDHA	6
17-HDHA	8
8-HEPE	8
9-HEPE	10
20-HDHA	7
16-HDHA	3
13-HDHA	13
11-HETE	17
10-HDHA	3
8-HETE	4
11-HDHA	17
7-HDHA	1
9-HETE	18
8-HDHA	63

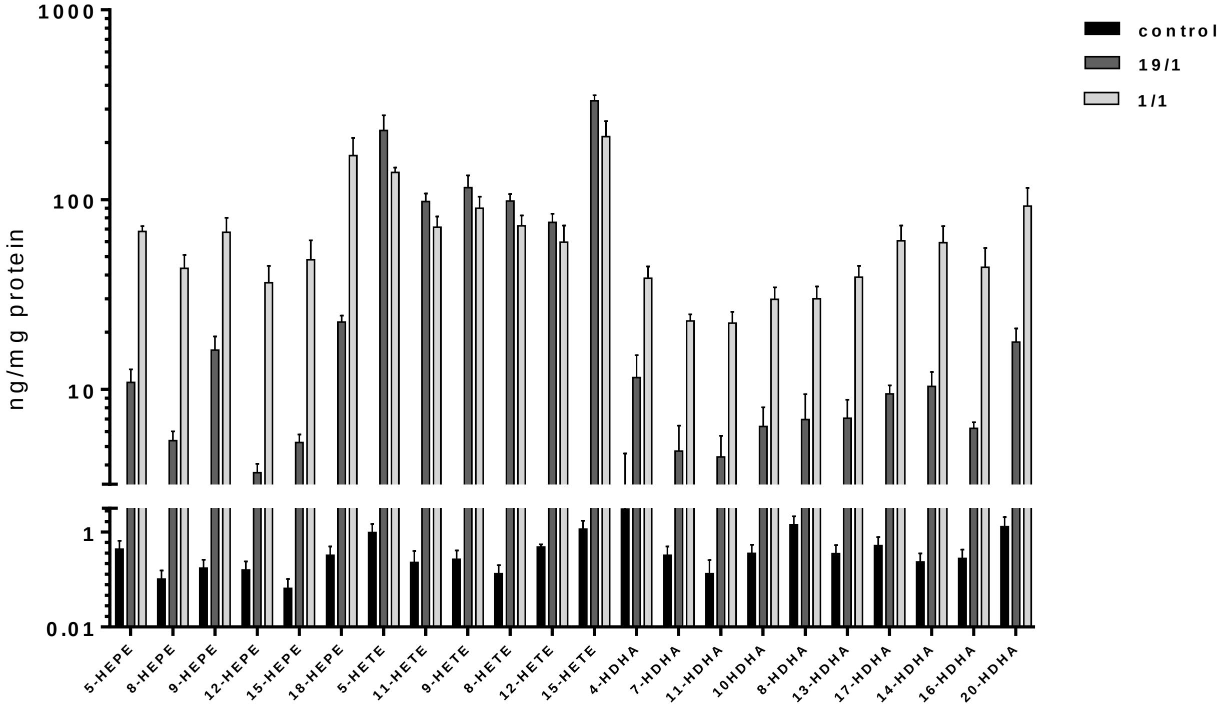

Figure S1. Monohydroxylated lipid metabolites in THP-1 macrophages incubated in control medium compared to the levels found with PUFA addition (ng/mg cell protein).

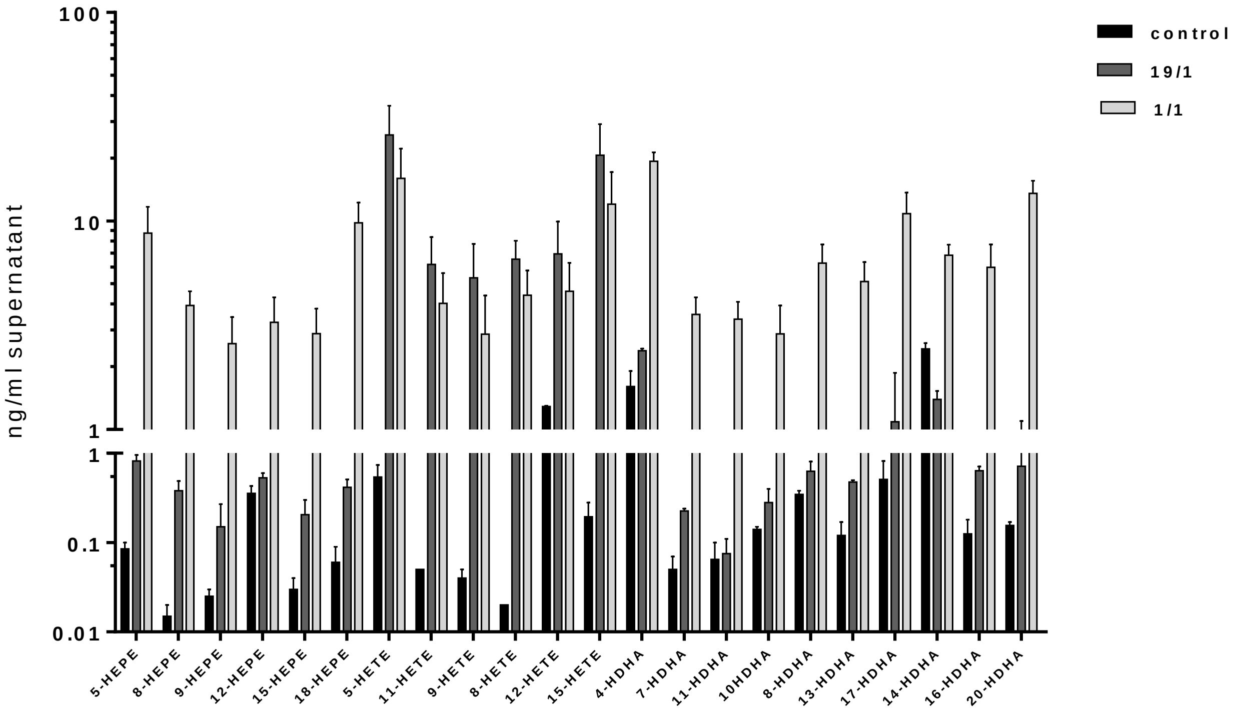

Figure S2. Baseline levels of lipid metaboiites: Cell-free incubations of tissue culture media containing the two different PUFA ratios were carried out, and compared to incubations of cell culture media without PUFA addition.
